# The Case of Insertional Adductor Tendinopathy of an International-Level 3,000-m Steeplechase Runner

**DOI:** 10.3389/fspor.2021.688280

**Published:** 2021-06-08

**Authors:** Ewan Thomas, Marcello Giaccone, Angelo Iovane, Gaspare Polizzi, Marco Petrucci, Giuseppe Messina, Antonio Palma

**Affiliations:** ^1^Sport and Exercise Research Unit, Department of Psychology, Educational Science and Human Movement, University of Palermo, Palermo, Italy; ^2^University of Palermo Sport Center (Centro Universitario Sportivo (CUS) Palermo), Palermo, Italy; ^3^Palermo Football Club, Palermo, Italy

**Keywords:** groin pain, tendinopathie, runner, athlete, malocclusion

## Abstract

**Background:** Groin pain is a frequent condition among athletes. One of the causes of groin pain is tendinopathy, a frequently diagnosed medical condition, which can also occur in the adductor muscles. Despite the high prevalence of this medical condition among athletes, it is infrequent to observe tendinopathic groin pain in steeplechase runners. The aim of this case study is to describe the case of an international-level 3,000-m steeplechase runner with groin pain, who was subsequently diagnosed with adductor insertional tendinopathy.

**Case Presentation:** We present the case of an Italian 3,000-m steeplechase and long distance runner, Ala Zoghlami (180 cm, 57 kg), with groin pain, diagnosed as insertional adductor tendinopathy. The runner, after manifesting the painful symptomatology, underwent medical screening (ultrasound and MRI). The radiological investigations highlighted adductor tendinopathy. After refraining from training, the runner underwent medical and physical therapy which, in the first phase, did not improve the painful symptomatology. Further evaluation, after 6 months from the initial training cessation, highlighted a case of malocclusion. Such was treated from a dentistry perspective with the creation of a personalized dental bite.

**Results:** A multidisciplinary approach which included medical and physical therapy, osteopathy, and dentistry, in adjunct with refraining from training, was able to reduce the symptomatology and allowed a correct return to run (after 9 months from the first painful manifestation) of the steeplechase runner. To date, Ala Zoghlami has fully recovered and was able to win the 3,000-m steeplechase race during the 2021 national Italian competition.

## Background/Context

Strenuous work-related activities, physical activity, and sports increase the risk of tendon overuse and the incidence of tendon injury (Andres and Murrell, 2008). Tendinopathy is a frequently diagnosed musculoskeletal clinical condition characterized by pain, swelling, and functional limitation of the tendon and the contiguous structures (Loiacono et al., 2019). Sport-related overuse tendinopathies are frequently diagnosed in the Achilles tendon in runners, in the patellar tendon in volleyball, handball, and basketball players, in the rotator cuff tendons in players of overhead sports, and in the extensor carpi radialis brevis tendon in tennis players (Ackermann and Renström, 2012).

A frequently diagnosed complication in sports in which rapid changes of direction repeatedly occur, such as football (Bisciotti et al., 2015), is groin pain which can also be associated to a tendinopathy (Weir et al., 2015).

A study evaluating a sample of 1,851 elite track and field athletes has reported that 249 injuries occurred during an international event. However, only 4.8% of these consisted of groin pain, the majority of which were related to strains and only in a confined number of cases associated to tendinopathies (Alonso et al., 2012). Nevertheless, the frequency of injuries reported in long distance runners and, in particular, in the 3,000-m steeplechase was of seven injuries, representing 2.8% of the total. Another survey conducted among track and field athletes regarding injury incidence among competitions has confirmed that very low injury rates are observed in the thigh or hip of long distance runners, and similar values are also reported in hurdle runners (D'Souza, 1994). Conversely, long distance runners have a tendency to develop low back pain and knee injuries (7 to 50% of cases) (van der Worp et al., 2015).

Weekly running distance and training frequency, previous history of injury, and a high number (more than six) of race participations a year are important risk factors associated to increased injury rate in runners (van der Worp et al., 2015). The diagnosis of adductor tendinopathy as a cause of groin pain is, however, infrequent also in non-sport populations (Albers et al., 2016), which is usually associated to pubic aponeurosis injury (Falvey et al., 2016). Such diagnosis may be performed in athletes with a previous history of groin pain along with pain or weakness of the adductor muscles and following an MRI investigation (Avrahami and Choudur, 2010; Mosler et al., 2015).

The management of tendinopathy usually involves the use of non-steroidal and steroidal anti-inflammatory medications, physical therapy, exercise therapy, and surgery (Andres and Murrell, 2008; Millar et al., 2021). Evidence also suggests that active exercise compared to passive treatment and the addition of manual therapy shorten the time to return to sport in athletes with history of groin pain (Serner et al., 2015).

Very little is known regarding insertional tendinopathy of the adductor muscles in elite athletes; therefore, we report the medical and training history of an international-level 3,000-m steeplechase runner presenting groin pain caused by insertional adductor tendinopathy.

## Case Description

The case here presented is that of an Italian 3,000-m steeplechase and long distance runner. Ala Zoghlami was born in Tunisia on June 19, 1994 (180 cm and 57 kg) and emigrated in Italy as a child. His career as a runner starts in 2004 at the age of 10. However, Ala Zoghlami started competing for the society CUS Palermo only in 2010. In 2013, he was able to obtain Italian citizenship, allowing him to be officially recognized by the Italian track and field federation (FIDAL) as an Italian runner. From this point, he starts competing at the national level and becomes a national youth champion for 3,000-m steeplechase in 2013, junior national champion in 2014 and 2015, and national absolute speciality champion in 2017. During the year 2017, he was called by FIDAL to represent the Italian national team at the IAAF world competition held in London for the 3,000-m steeplechase, which he closes at 8:26.18 in 16th position.

In February 2019, during a training session, Ala Zoghlami starts feeling groin pain in the left leg, which manifests only during the jumping phase of the 3,000-m steeplechase.

The methodological and medical approach to the problem is discussed in the following section.

## Methodology/Approach To The Problem

**Figure 1:**
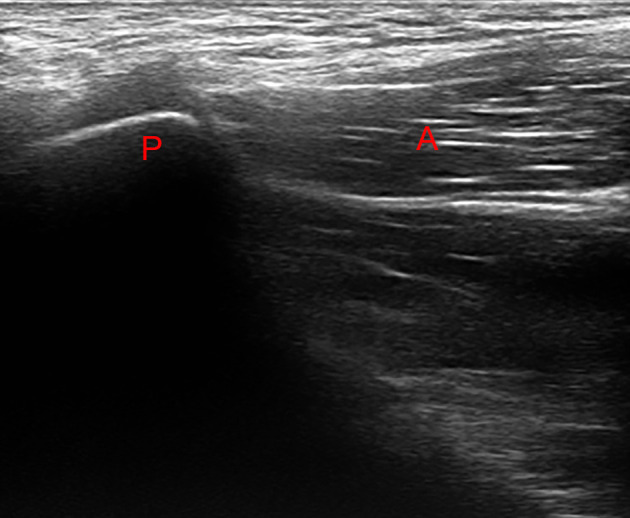
Ultrasound investigation showing pubic insertion of the adductor musculature. No evidence of functional overload is present.

**Figure 2:**
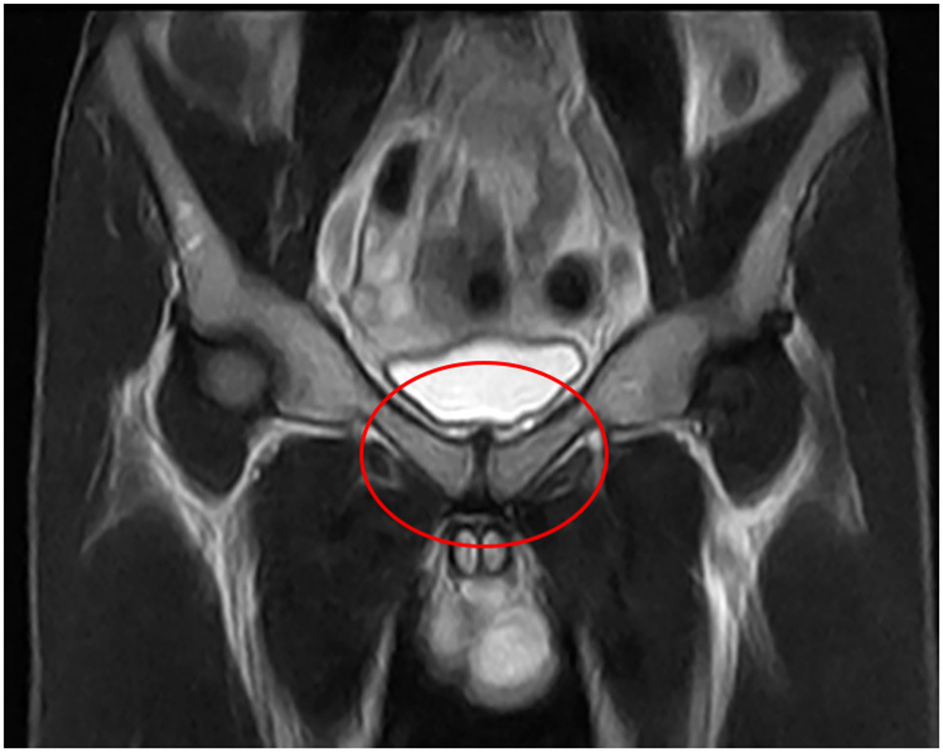
MRI investigation of the frontal plane in T2W, in which no functional overload of the pubic area is present.

After 7 days of pain onset, within the period when the runner continued his training regime with moderate pain intensity, the coach and training staff decided to investigate the reason of the painful situation. An ultrasound investigation and magnetic resonance imaging (MRI) was performed, and at first examination no evident medical condition was diagnosed (Figures 1, 2).

**Figure 3:**
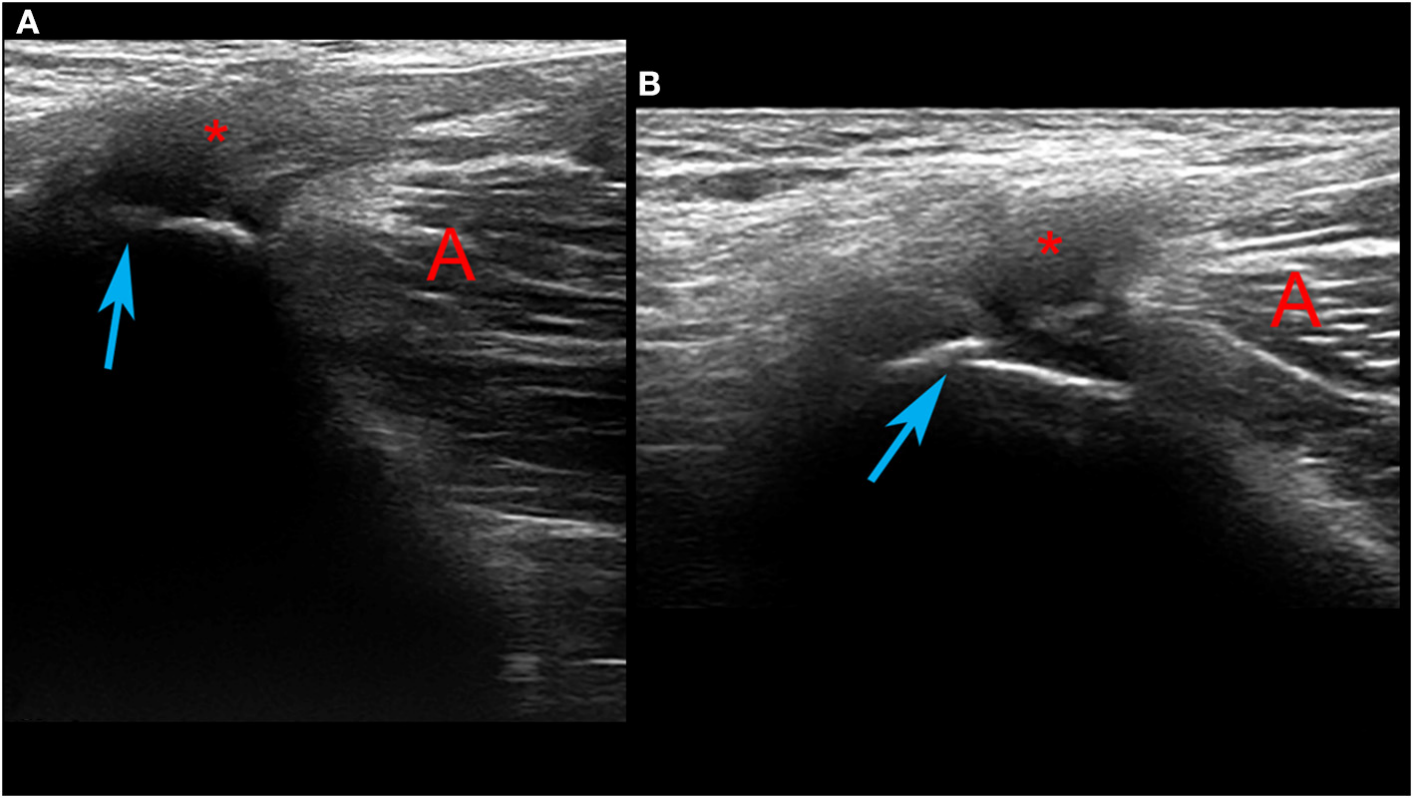
Ultrasound investigation of the pubic area. Functional overload of the adductor insertion (marked with an asterisk) is shown, with an irregularity of the superficial cortical profile (blue arrow) of the pubis. Sections **(A,B)** provide different planes of view of the pubic functional overload.

**Figure 4:**
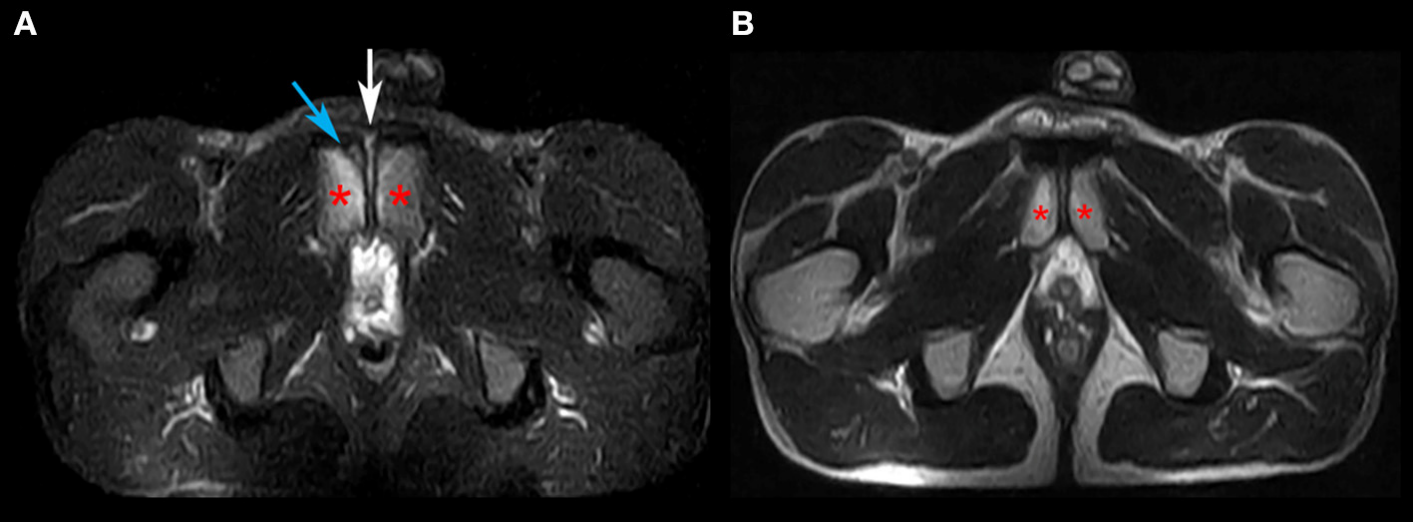
MRI investigation on the axial plane. **(A)** STIR. **(B)** T2W. Images highlight the irregularity of the superficial cortical profile identifying osteochondral dissociation of the area (blue arrow), with coexistent endo-articular hyperemia (white arrow) and light subchondral bone hyperemia (left). The pubis is marked with an asterisk.

Following the medical examinations, as a precautionary approach, the runner refrained from training for 10 days in order to decrease the symptomatology, thinking that the functional overload was caused by training. After another 10 days of reconditioning training (20 days from the start of medical investigations), a time during which no jumps were performed during the training sessions, the runner regains training in 3,000-m steeplechase and, after his first jump, the painful symptomatology re-presents bilaterally. On the following day, first an ultrasound and then an MRI investigation, which was conducted to confirm the ultrasound findings, were performed, diagnosing insertional tendinopathy of the adductor muscles (Figures 3, 4).

**Figure 5:**
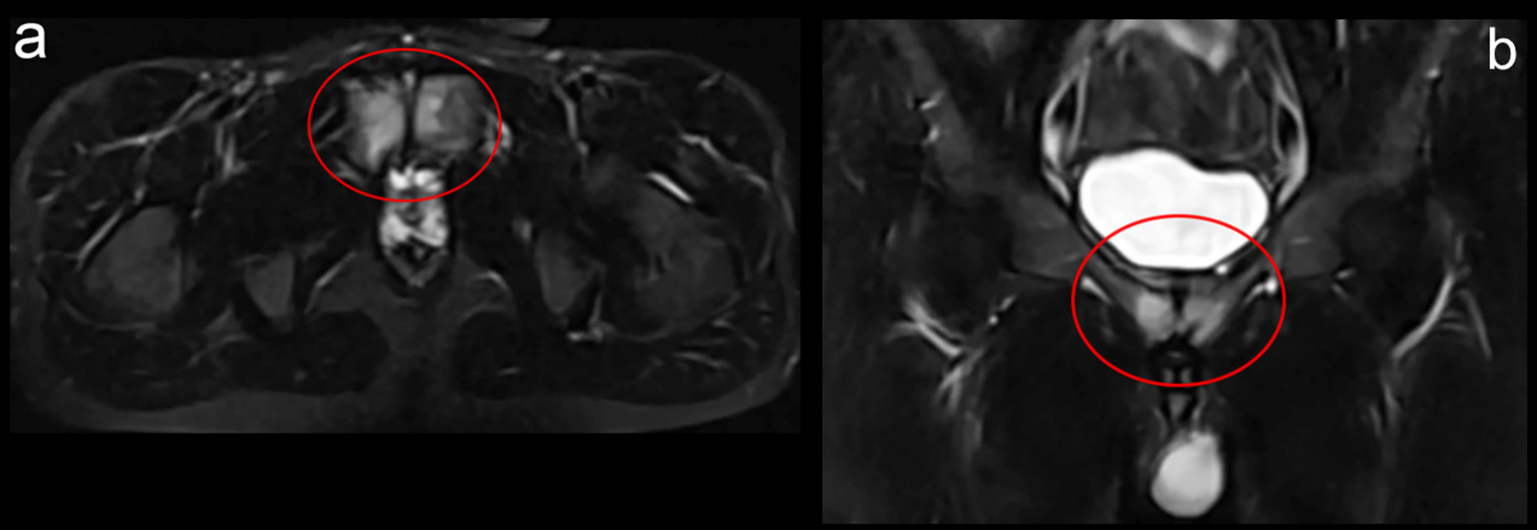
MRI investigation on the axial **(a)** and coronal plane **(b)**. The exam highlights an increase of the phlogistic phenomenon of the pubic symphysis compared to Figure 4.

Notwithstanding the painful symptomatology, Ala Zoghlami had to participate at the beginning of March 2019 for a period of 20 days with the FIDAL national team at special altitude training sessions in Flagstaff, Arizona, USA. During this period, upon medical advice, the runner takes non-steroidal anti-inflammatory drugs (NSAIDs) for 1 week. However, no difference in the symptomatology occurred. In March 2019, a third MRI investigation was performed as the symptomatology worsened after the altitude training session(Figure 5). The MRI highlighted bone hyperemia on the pubic symphysis, which was observed bilaterally with irregularities of the superficial cortical profile. It was concluded that there was an increase of the phlogistic phenomenon of the adductor muscles reflecting on the bone. As a consequence of the MRI findings, from the end of March 2019 (after returning in Italy) to December 2019, the runner completely refrained from training. In order to improve the clinical condition, from April to September the athlete undergoes physical therapy. This was provided at a frequency of two treatments per week, consisting of sessions of manual therapy directed to the lumbar spine, pelvis, and lower limb and other treatments such as capacitive resistive electric transfer and low-level laser therapy when deemed necessary by the physical therapist. Despite the physical therapy provided, as with the NSAIDs, the runner was not able to fully recover and return to run. At this point, in September 2019, despite the fact that the pain intensity decreased, this was still present, and Ala Zoghlami was still not able to undergo a training regime. Therefore, the athlete's coach decided for him to consult an osteopath for further evaluation and treatment. The consulted osteopath, following the manual therapy, highlights a possible occlusal dysfunction and therefore suggests a consultation with a dentist.

The subsequent dental evaluation confirms an occlusal and temporomandibular dysfunction (a click was evaluated on the right temporomandibular joint during mouth opening) and identifies the need to use a dental bite. The occlusal dysfunction identified was a class II first division. Dental impressions were taken in alginate, and a centric position was detected, advancing the mandible by 2 mm. A wax model was subsequently sent to a laboratory for the creation of a semi-rigid bite. The type of bite adopted was inferior (mandibular). The inferior bite was used in order to allow a correct tongue position on the posterior aspect of the superior jaw during swallowing (Messina, 2020) (parte sul titan). After 1 week of wearing the bite by the athlete, he referred that the symptomatology of the groin area decreased. Since the symptomatology result decreased, it was opted for him to not undergo immediate medical screening.

**Figure 6:**
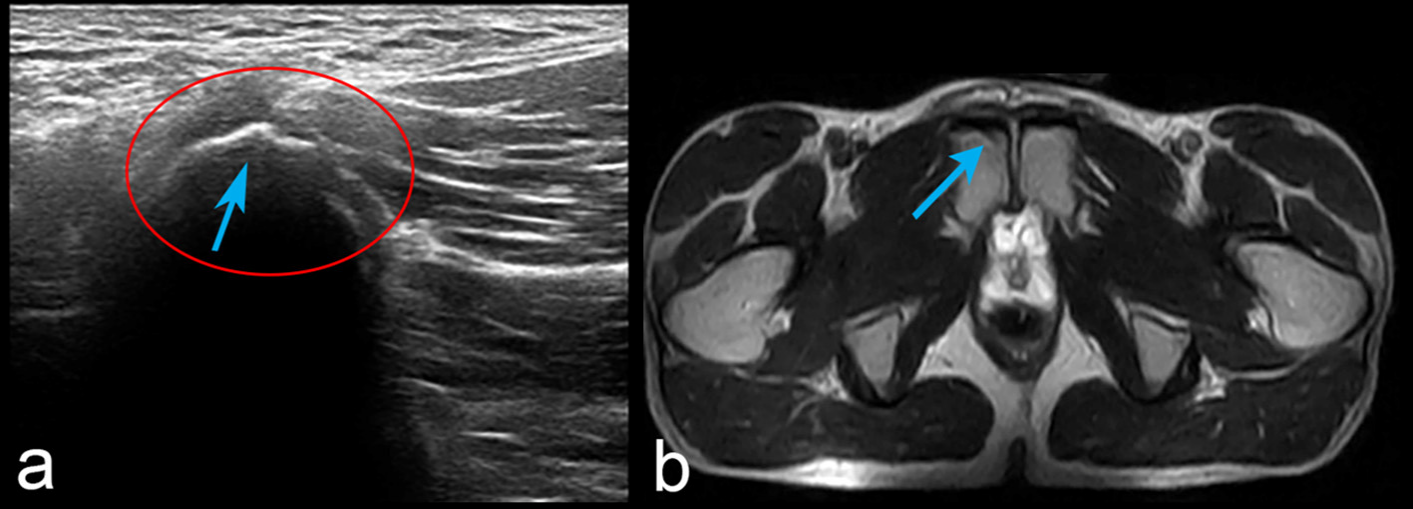
Ultrasound **(a)** and MRI investigation on the axial plane **(b)**. Both images highlight a reduction of the phlogistic phenomenon (blue arrow). The cortical profile irregularity, however, remains.

Notwithstanding the reduction of the painful symptomatology, the coach, the team, and FIDAL decided for a precautionary approach and encouraged the athlete to refrain from training up to January 2020. In December 2019, Ala Zoghlami underwent a final ultrasound and MRI investigation, which highlighted a reduction of the tendinopathic condition (Figure 6) with almost absence of pain of the groin area.

To date, Ala Zoghlami has fully recovered and still uses the dental bite before, during, and after training and competitions. In August 2020, he qualified in second position at the Italian national competition in the 3,000-m steeplechase (8:26.22) and won the 5,000-m race (14:04.44).

## Discussion

With the present case study, we report the clinical manifestation of an adductor insertional tendinopathy in a steeplechase runner of international level. The medical records, together with the treatment approach adopted, highlight that despite the fact that such typology of injury is rarse in its presentation (Alonso et al., 2012), there is a possibility for the athlete to recover. In our case, we adopted a multidisciplinary approach (involving medical and physical therapy, osteopathy, and dentistry) which allowed the runner to eventually return to run.

When particularly complicated situations or when failure of standard care occurs, multidisciplinary approaches involving different professionals may be advisable in order to guarantee better clinical outcomes, especially in situations of chronic pain (Scascighini et al., 2008). It is important for us to highlight that we had initially failed to recover and correctly determine which approach could have been more beneficial for our athlete in order to fully recover from the injury. Therefore, we suggest to not underestimate pain in such an uncommon population. Similar to our athlete, another case study (Feigenbaum et al., 2016) of a college football player, who fractured the lateral malleolus, reports that a multidisciplinary approach was able to successfully and safely enable the athlete to return to his sport, avoiding excessive deconditioning.

Since interdisciplinary approaches are frequently warranted and osteopathy, in particular, can be applied for different conditions (Tarsuslu et al., 2009; Cerritelli et al., 2013; Orrock and Myers, 2013), we decided, following a long period of physical therapy and rest, to consult an osteopath who, after a careful analysis, suggested the potential presence of a malocclusion. Despite that there is still conflicting evidence regarding the association between osteopathy and dentistry (Andresen et al., 2013), in particular, regarding osteopathic treatment and improvement of malocclusion, a subsequent dental visit was warranted.

It is still not clear how come the symptomatology was reduced after the dental bite was used. In addition, it is important to underline that a very long resting period passed from the initiation of the symptoms to complete resolution, with four different approaches adopted during this timeframe. However, it has to be noted that the study of Michelotti et al. (2011), evaluating possible associations between occlusion and posture, has found some associations between occlusal dysfunctions and postural alterations. In particular, the authors highlight the neuroanatomical connections between the trigeminal nuclei which innervate the temporomandibular joint and both vestibular and cervical nuclei. Such connections may imply that a malocclusion may modify the head's posture and vestibular control. The authors also focused on the possible association between crossbites and scoliosis, which could first explain how come a malocclusion could lead to a modification of the pubic morphology and secondly explain the reduction of the pubic alterations following the application of the dental bite. Therefore, according to these findings, a reduction of the malocclusal asymmetry could have led to a reduction of the mechanical forces applied to the pelvis. Another investigation by Sambataro et al. (2019) has also discussed the implications of postural assessment in situations of temporomandibular dysfunctions, stressing that correlations between posture and mandibular dysfunctions are present, highlighting the need of multidisciplinary approaches in order to correctly identify and treat these subtle conditions. Despite the interesting considerations discussed by Michelotti et al. (2011) and Sambataro et al. (2019) it is still premature to conclude that a pathological condition observed in the pelvis is caused by an occlusal dysfunction.

**Figure 7:**
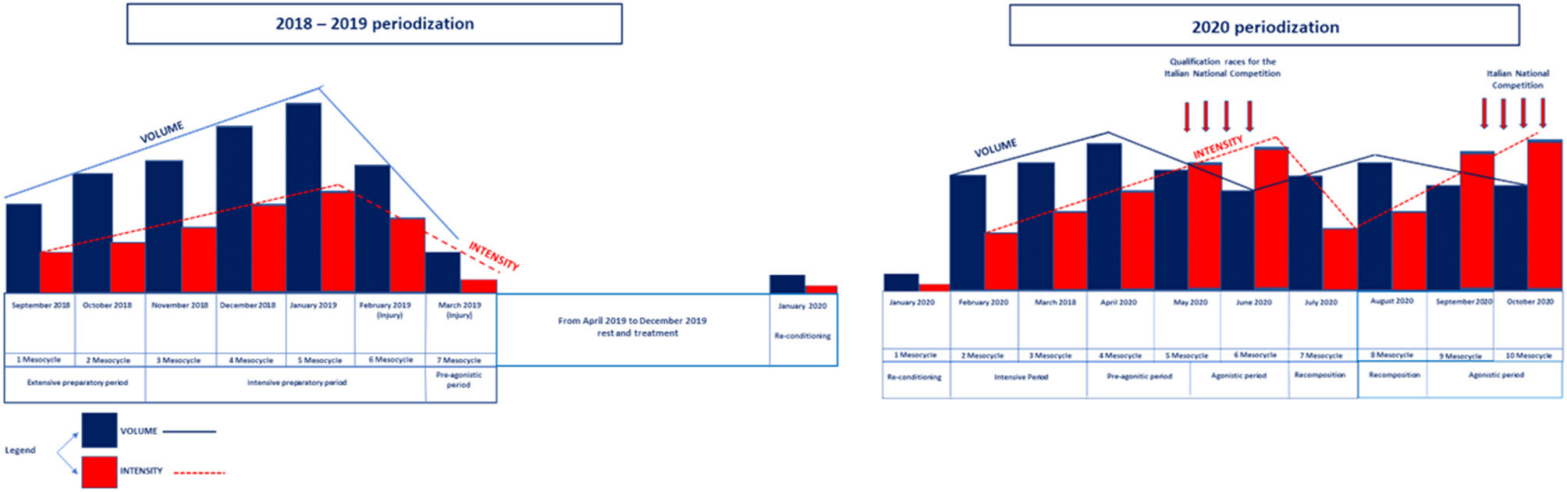
Training periodization before and after the injury. As shown in this figure, Ala Zoghlami adopted an increasing volume and intensity training regimen in the years 2018 and 2019. Subsequently, in March 2019, he had to refrain from training up to January 2020. The second part of the periodization shows the year 2020 which highlights a double periodization, with increased and decreased volume and training intensity which were adopted in order to not overload the athlete, leading to a full recovery and the victory of a medal [August 2020, second place at the Italian national competition in the 3,000-m steeplechase (8:26.22) and first place in the 5,000-m race (14:04.44)].

Despite that the symptomatology was reduced following this multidisciplinary approach, Ala Zoghlami refrained from training for almost 9 months, which is a very long period for a professional athlete. In addition, another period following the injury was needed in order to fully regain the athletic capabilities (Figure 7). However, following the above-described approach, Ala Zoghlami was eventually able to recover and win a competition at a national level first in 2020 and then in 2021.

Our suggestion, based on this clinical presentation, is to not underestimate pain in professional athletes and to manage the pain and recovery of this particular population based on a multidisciplinary approach, also including non-conventional forms of treatment if the ratio between risks and benefits may lead to a clinical advantage.

## Take-Home Message

1. Adductor tendinopathy is infrequent in steeplechase runners.
2. Multidisciplinary approaches should be immediately undertaken in order to reduce the time to return to run.
3. Non-conventional treatment in the case of persistent and recurrent pain may provide additional therapeutic support.

## Data Availability Statement

The original contributions presented in the study are included in the article/supplementary material, further inquiries can be directed to the corresponding author/s.

## Ethics Statement

Written informed consent was obtained from the individual(s) for the publication of any potentially identifiable images or data included in this article.

## Author Contributions

ET: writing and review. MG, MP and GP: methodology. AI: formal analysis. GM: resources. AP: writing and review and supervision. All authors contributed to the article and approved the submitted version.

## Conflict of Interest

The authors declare that the research was conducted in the absence of any commercial or financial relationships that could be construed as a potential conflict of interest.
